# Mild to moderate drought stress reinforces the role of functional microbiome in promoting growth of a dominant forage species (*Neopallasia pectinata*) in desert steppe

**DOI:** 10.3389/fmicb.2024.1371208

**Published:** 2024-05-22

**Authors:** Hui Gao, Zhenzhen Huang, Weiwei Chen, An Xing, Shixiang Zhao, Weifan Wan, Haina Hu, Haigang Li

**Affiliations:** Inner Mongolia Key Laboratory of Soil Quality and Nutrient Resources, Key Laboratory of Agricultural Ecological Security and Green Development at Universities of Inner Mongolia Autonomous Region, College of Grassland, Resources and Environment, Inner Mongolia Agricultural University, Hohhot, Inner Mongolia, China

**Keywords:** synthetic microbial community, natural grassland, *Neopallasia pectinata*, stress resistance, plant growth

## Abstract

**Background:**

Desert steppe ecosystems are prone to drought stress, which influences the ecological balance and sustainable development of grasslands. In addition to directly restrict plant growth, drought stress indirectly impacts plant fitness by altering the diversity and function of root-associated microbiomes. This begs the question of whether the functional microbiome of forage plants, represented by synthetic microbial communities (SynComs), can be leveraged to mitigate drought stress in desert steppes and promote the ecological restoration of these fragile ecosystems.

**Methods:**

A pot experiment was conducted to evaluate the role of SynComs in improving the plant growth and drought stress resistance of *Neopallasia pectinata* (Pall.) Poljak in desert steppe in Inner Mongolia, China. Six SynComs were derived from the rhizosphere and root endosphere of 12 dominant forage species in the desert steppe. Each SynCom comprised two to three bacterial genera (*Bacillus*, *Protomicromonospora*, and *Streptomyces*). We examined the capacities of different SynComs for nutrient solubilization, phytohormone secretion, and enzymatic activity.

**Results:**

Under no water stress (75% soil water holding capacity, WHC), single strains performed better than SynComs in promoting plant growth in terms of stem diameter, root length, and plant dry weight, with the greatest effects observed for *Streptomyces coeruleorubidus* ATCC 13740 (*p* < 0.05). However, under mild to moderate drought stress (55% and 35% WHC), SynComs outperformed single strains in enhancing plant biomass accumulation and inducing the production of resistance-related substances (*p* < 0.05). No significant effect of single strains and SynComs emerged under extreme drought stress (20% WHC).

**Conclusion:**

This study underscores the potential of SynComs in facilitating forage plants to combat drought stress in desert steppe. Mild to moderate drought stress stimulates SynComs to benefit the growth of *N. pectinata* plants, despite a soil moisture threshold (21% WHC) exists for the microbial effect. The use of SynComs provides a promising strategy for the ecological restoration and sustainable utilization of desert steppes by manipulating the functional microbiome of forage plants.

## 1 Introduction

Desert steppe is an ecotone from grassland to desert, with productivity limited by low soil moisture resulting from unique environmental and climatic conditions (Wu et al., 2010). Desert steppe regions are growing problems of drought stress due to global climate change and anthropogenic activity. Drought stress impairs the ecological function of desert steppes by changing the root morphology and structure of forage plants, retarding their growth rates, and reducing plant biomass (Huang et al., 2014; Kou et al., 2022). While desert steppes have fewer forage species than other grassland types, most of the dominant forage species are more susceptible to environmental stress because of their high individual abundance and coverage (Wang et al., 2002). Therefore, increasing drought stress resistance in the dominant forage species is essential for productivity improvement, ecosystem stabilization, and land restoration in desert steppes.

The survival and adaptability of drought-stressed forage plants can be enhanced by a range of methods, such as altering the morphology and structure of plant roots and increasing the accumulation of metabolites involved in plant stress resistance (Xie et al., 2011). Generally, exogenous hormones are used to modulate plant growth, and as such, improve water use efficiency. Biological amendments modify soil structure and increase water retention capacity, indirectly assisting plants to withstand drought stress. Additionally, plant growth-promoting rhizobacteria facilitate plant absorption of water and nutrients through nitrogen (N) fixation as well as phosphorus (P) and potassium (K) solubilization (Gupta et al., 2020; Meena et al., 2021). In particular, the rhizosphere microbiome, which is regarded as the plant’s second genome (Shen et al., 2021), relates closely to the growth and development of host plants. Due to their outstanding stress resistance, microbial communities maintain relatively stable structure and function after perturbations (Bardgett and Caruso, 2020). Therefore, the rhizosphere microbiome acts as an indispensable driver of plant growth and an essential contributor to ecosystem function.

There are mainly two mechanisms by which the rhizosphere microbiome promotes plant growth and alleviates plant stress. The first mechanism is promotion of plant root growth and development; this is exemplified by microbial solubilization of mineral nutrients and production of phytohormones (e.g., indole-3-acetic acid, IAA) to alter root structure and morphology. The second mechanism is enhancement of plant tolerance to abiotic stresses by upregulating their biosynthesis of endogenous hormones (e.g., abscisic acid, ABA) and accumulation of osmoregulators (e.g., proline, PRO; Mayak et al., 2004; Yang et al., 2009). The ability of rhizosphere microbiome to bolster plant resistance and resilience is variable under different drought stress conditions. For instance, plant growth-promoting rhizobacteria play a potential role in alleviating drought tolerance in foxtail millet (*Setaria italic* L.) grown in a semi-arid agroecosystem (Niu B. et al., 2017). In the case of blue grama (*Bouteloua gracilis*), a dominant grass species in semi-arid grassland ecosystems, microbial inoculation positively affects plant performance under well-watered or moderate drought conditions, whereas negative effects emerge during extreme drought (Ulrich et al., 2019). The possible reason is that low drought stress reduces the bacterial and fungal diversity while increasing the network complexity of soil microbiomes (Li et al., 2023); intense drought destabilizes the microbiome network, reducing its role in plant growth (De Vries et al., 2018).

Previous findings indicate that the rhizosphere microbiome may have limitations in improving plant growth and drought stress resistance. In natural grasslands, the soil moisture threshold at which microbial communities dramatically shift is 15% water holding capacity (WHC; Cordero et al., 2023). Myriad studies have looked at the structure of rhizosphere microbiomes and their functional role in improving soil health in agroecosystems with single vegetation types and intense artificial perturbations (Niu X. et al., 2017; Chandra et al., 2019; Li et al., 2024). In contrast to agroecosystems, natural ecosystems are more resilient and resistant to environmental stresses owing to complex plant–soil–microbe interactions. Currently, there is still little solid evidence for the effects of rhizosphere microbiomes on drought-stressed plants under natural conditions, especially in unique desert steppe ecosystems. How the functional microbiome of forage plants affects plant growth and drought stress resistance has not been reported in desert steppes, and the underlying mechanisms remain elusive. Research is also necessary to ascertain whether there exists a soil moisture threshold for the beneficial effects of the functional microbiome on forage plants in desert steppe regions.

While a single strain has limited adaptability, stability, and efficiency, synthetic microbial communities (SynComs) have multiple advantages. Many SynComs are developed using a reduced number of microbes from the rhizosphere of plants to mimic the structure and function of natural microbiomes. By reducing the complexity of SynComs, the functional role of community members is maximized (Gu et al., 2020; Xia et al., 2022). Here, we developed SynComs by combining three distinct bacterial genera isolated from the rhizosphere and root endosphere of dominant forage species in the desert steppe of Inner Mongolia, China. The potential of SynComs in promoting plant growth and drought stress resistance was determined by analyzing the metabolic functions of microbial consortia. One of the dominant forage species in the study area is *Neopallasia pectinata* (Pall.) Poljak, a herbaceous plant of the family Asteraceae that is either annual or biennial. Therefore, we investigated the effects of SynComs on the plant growth and drought stress resistance of *N. pectinata* under various stress conditions. The aim of this study was to unravel the intertwined connections between the functional microbiome, soil properties, and dominant forage traits in desert steppe. The results could be useful to provide new strategies for the ecological restoration and sustainable management of desert steppe. We hypothesized that compared with single strains, SynComs perform better in colonizing the rhizosphere, benefiting biomass accumulation, and improving drought stress resistance of forage plants; these beneficial effects are likely to be dampened and even reversed under extreme drought conditions.

## 2 Materials and methods

### 2.1 Strains, soil, and seeds

We selected five strains of rhizosphere and root endosphere bacteria (B1, B2, and A1–A3) preserved in the Inner Mongolia Key Laboratory of Soil Quality and Nutrient Resources, Inner Mongolia Agricultural University (Hohhot, Inner Mongolia, China). These strains were isolated and screened from 12 dominant forage species at the field experiential base in Adege (Siziwang Banner, Ulanqab, Inner Mongolia): *Stipa breviflora* Griseb., *Cleistogenes songorica* (Roshev.) Ohwi, *Medicago ruthenica* (L.) Trautv., *Calystegia sepium* (L.) R. Br., *Allium tenuissimum* L., *Allium polyrhizum* Turcz. ex Regel, *Neopallasia pectinata* (Pall.) Poljak., *Artemisia frigida* Willd., *Asparagus cochinchinensis* (Lour.) Merr., *Aster altaicus* Willd., *Leymus chinensis* (Trin. ex Bunge) Tzvelev, and *Erodium stephanianum* Willd.

The basic information of the tested strains is provided in Table 1. Strains were isolated and purified using beef extract peptone medium and Gauze’s synthetic medium No. 1 (Bertani, 1951; Bai, 2020). Based on the sequencing results of the 16S rDNA gene, these bacterial isolates belonged to 3 genera. These strains were capable of solubilizing P and K as well as producing phytohormones and 1-aminocyclopropane-1-carboxylate deaminase (ACCD; unpublished data). We employed a ‘bottom-up’ approach for reductionist combination of the five strains (Niu B. et al., 2017). A total of six SynComs (one full five-member community: G1 and five dropout communities: G2–G6) were constructed (Table 1).

**Table 1:** Origin of functional strains, their closest species, and design of synthetic microbial communities.

Origin	Closest species	Type strain	Sequence similarity	CCTCC accession no.	Combinatorial approach					
					G1	G2	G3	G4	G5	G6
Root endosphere	*Bacillus* sp. M3_1	MG758004.1	100.00%	CCTCC M 20231311	B1	B1	B1	B1	B1	-
Root endosphere	*Bacillus* sp. DE030	KY860723.1	100.00%	CCTCC M 20231312	B2	B2	B2	B2	-	B2
Root endosphere	*Promicromonospora* sp. JCM 28668	LC133699.2	99.38%	CCTCC M 20231313	A1	A1	A1	-	A1	A1
Rhizosphere	*Streptomyces peucetius* subsp. *caesius* ATCC 27952	CP022438.1	100.00%	CCTCC M 20231314	A2	A2	-	A2	A2	A2
Rhizosphere	*Streptomyces coeruleorubidus* ATCC 13740	CP023694.1	99.70%	CCTCC M 20231315	A3	-	A3	A3	A3	A3

Soil and *N. pectinata* seeds were collected from the field experimental base in Adege Siziwang Banner, Ulanqab, Inner Mongolia (42°02′17′′ N, 112°30′57′′ E), at an altitude of 1,327 m. The soil type was classified as light chestnut soil, which had a pH of 8.45 and contained 2.45 mg kg^−1^ Olsen-P, 208.63 mg kg^−1^ NH_4_OAc-exchangeable K, 1.17 mg kg^−1^ nitrate-N, and 1.05 mg kg^−1^ ammonium-N. The collected soil was air-dried, sieved (2 mm), and sterilized (160°C–170°C, 2 h) before use.

### 2.2 Metabolic function analysis

*Bacillus* strains (B1 and B2) were grown in beef extract peptone medium, and *Promicromonospora* and *Streptomyces* strains (A1, A2, and A3) were grown in Gauze’s synthetic medium No. 1 (Bai, 2020). All liquid cultures were incubated at 28°C with oscillation at 180 r min^−1^ for 2 d (B1 and B2) or 7 d (A1, A2, and A3). After that, the cultures were collected and mixed in equal volumes based on the combinatorial approach in Table 1. The combined cultures (G1–G6) were stored at 4°C until use.

We inoculated 10.0 mL each of combined cultures into Pikovaskaia’s inorganic P medium, Monkina’s organic P medium, and K solubilizing medium without agar (Sun et al., 2017; Zhang et al., 2022). Autoclaved culture media without inoculation served as blank controls. After 7 d of incubation (28°C, 180 r min^−1^), the resultant cultures were centrifuged and filtered to obtain cell-free supernatants. The soluble P concentration in all supernatants was quantitatively analyzed by molybdenum antimony anti-colorimetric method (Feng, 2021). Flame photometry was employed to determine the soluble K concentration in supernatants (Sun et al., 2017), and K solubilization efficiency was calculated based on Eq. (1). Each treatment was repeated three times.


(1) $$K\;\mathrm{solobilization efficiency}=\frac{X_{1}-X_{0}}{M\times W\times 2\times 10^{4}}\times 100\%$$

where *X*_1_ is the soluble K concentration of samples inoculated with combined cultures (mg L^−1^); *X*_0_ is the soluble K concentration of samples in the blank control (mg L^−1^); *M* is the mass of K-feldspar (g); *W* is the K content of K-feldspar (*W =* 12%); and 2 × 10^4^ was used to convert the mass of K to its proportion in the samples.

The combined cultures were centrifuged at 4°C and 7,104 × *g* for 10 min and filtered through 0.22-μm microporous membranes (Beckman Coulter, Shanghai, China). The filtrates were collected and sent to Norminkoda Biotechnology Co., Ltd. (Wuhan, China), where auxin (indole-3-acetic acid, IAA) and cytokinin (CTK) concentrations and ACCD activity were measured by enzyme-linked immunosorbnent assay (ELISA).

### 2.3 Pot experiment design

#### 2.3.1 Experiment I

Evaluating plant growth promotion by functional microbiome under different water conditions.

The experiment used a two-factor completely randomized design: drought stress and microbial inoculation. The water conditions were set based on previous study (Hsiao, 1973) and preliminary experiment (Supplementary Figure S1): no stress (irrigation amount = 75% WHC) and drought stress (35% WHC). With regard to microbial inoculation, 12 treatments were established. There were one control group (autoclaved culture media) and 11 microbial treatment groups, including five single-strain treatments (B1, B2, A1, A2, and A3) and six SynCom treatments (G1–G6). Each treatment had three replications. Before the experiment started, we conducted a plate confrontation test to verify whether the five strains were antagonistic to each other. No antagonistic circles were produced on the plates, indicating the absence of antagonism.

Air-dried soil (1.00 kg) was filled into surface-disinfected pots with an upper diameter of 16 cm, a lower diameter of 9 cm, and a height of 10 cm. The cultures (10.0 mL each) of single strains or SynComs were inoculated to the soil at the time of irrigation with sterile water. Plump seeds were selected and pre-soaked in pure or mixed cultures for 24 h before sowing (eight per pot). All pots were placed in an artificial climate chamber and incubated for 7 days under the following conditions: temperature, 25°C; illumination time, 8:00–20:00; and light intensity, 400–600 μmol/(m^2^ s). Then, four seedlings with uniform growth were established in each pot and exposed to drought stress. A second inoculation (10.0 mL) was performed 20 days after sowing. Plants were harvested after 45 days of incubation and samples were collected to measure plant growth and physiological parameters, rhizosphere colonization by functional microbes, and soil physicochemical properties.

#### 2.3.2 Experiment II

Evaluating plant growth promotion by functional microbiome under different drought stress levels.

Experiment 1 only reflects the growth-promoting and water-stress-resistant effects of single strains and combinations under no water stress and water stress, the combination has a better effect under water stress. We consider the growth conditions of plants under different stress gradients designed in the pre-experiment to further evaluate the role of functional bacteria in promoting plant growth under different drought stress levels. The experiment also consisted of two factors: drought stress and SynCom composition. There were two treatments for drought stress, i.e., mild stress (irrigation amount = 55% of WHC) and extreme stress (20% of WHC). Considering SysComs, five treatments were established, i.e., one control group (autoclaved culture media), two single-strain treatment groups (strains that had the greatest and least effects on the relative growth rate (RGR) of plant biomass under drought stress in Experiment I), and two SynCom treatment groups (the full community G1 and the dropout community that had the greatest effect on RGR in Experiment I). The experimental procedures were the same as those used in Experiment I. Plant biomass was measured after harvest and compared with that under no stress (75% WHC) and moderate stress (35% WHC) conditions in Experiment I. The soil moisture threshold for the beneficial effects of functional microbiome on plant biomass was identified.

### 2.4 Sampling and measurement

Whole plant samples were collected at harvest, with the complete root system excavated. Rhizosphere soil samples were collected as previously described (Fan et al., 2017). Soil loosely adhering to the root surface (bulk soil) was gently shaken off and air-dried for the determination of soil physicochemical properties. Soil that was retained on the roots (rhizosphere soil) was transferred into cryogenic vials using a sterile brush and immediately stored at −80°C for the analysis of rhizosphere colonization by functional microbes.

A straightedge was used to measure root length as the vertical distance from the basal part of the stem to the lowermost part of the root (mm), and a Vernier caliper was used to measure stem diameter at the basal part of the stem (mm). Afterwards, the roots were rinsed with deionized water and scanned using a root scanner (Perfection V850 Pro; Epson, Suwa, Japan). The acquired images were processed using Adobe Photoshop CC (version 2019; Adobe Systems, San Jose, CA, United States). Subsequently, whole plant samples were deactivated at 105°C for 30 min and then oven-dried at 65°C until constant weight. The total biomass (g plant^−1^) was measured and the RGR under drought stress (Experiment I) was calculated for different treatment groups according to Eq. (2):


(2) $$\begin{array}{l} \mathrm{RGR}\;\left(\%\right)=\\ \frac{\begin{array}{l} \mathrm{Biomass of stressed plants}-\\ \mathrm{biomass of non}-\mathrm{stressed plants} \end{array}}{\mathrm{Biomass of non}-\mathrm{stressed plants}}\times 100\% \end{array}$$

Three plants per replication were snap-frozen in liquid nitrogen and stored at −80°C. The frozen samples were sent to Norminkoda Biotechnology Co. Ltd. for the measurement of endogenous hormone contents. PRO content was determined by sulfosalicylic acid-indanedione method using a commercial kit (NMKD0105; Norminkoda Biotechnology Co., Ltd.). To quantify abscisic acid (ABA) content, plant tissues were ground with liquid nitrogen and a 0.1-g sample was extracted with 1.0 mL of normal saline by ultrasonication in an ice-water bath for 30 min. The extracts were analyzed using an ELISA kit (NM1205; Norminkoda Biotechnology Co., Ltd.).

The MagPure Soil DNA LQ Kit (Magan, Shanghai, China) was used to extract genomic DNA from rhizosphere soils as per the manufacturer’s instructions. After the purity and concentration were checked, DNA samples were sent to Oebiotech (Shanghai, China) for bacterial 16S rRNA (V3–V4) amplicon sequencing, which was accomplished on the Illumina NovaSeq 6000 platform (Illumina Inc., San Diego, CA, United States). As the five strains were classified into the genera *Bacillus*, *Promicromonospora*, and *Streptomyces*, we selected the relative abundance data (expressed as percentages) of these three taxa from sequencing analysis to evaluate rhizosphere colonization.

Bulk soils were passed through a 2-mm sieve before the analysis of nutrient availability and pH. Soil pH measurements were conducted in suspensions with a soil-to-water ratio of 1:5 (w/v) using a DZS-706F multiparameter meter (Leici, Shanghai, China). Soil Olsen-P content was determined by molybdenum antimony anti-colorimetric method after sample extraction with NaHCO_3_-NaOH solution. Soil exchangeable K content was determined by flame photometry after extraction with NH_4_OAc (Bao, 2000).

### 2.5 Statistical analysis

All data were analyzed using Microsoft Excel (version 2019; Microsoft Corp., Redmond, WA, United States) and SAS (version 8; SAS Institute Inc., Cary, NC, United States). The RStudio software (version 4.2.1; R Core Team, 2022) was adopted to create the bubble plot of taxa relative abundance and the matrix of Spearman’s correlation coefficients, as well as bar charts, box plots, and trend lines. One-way analysis of variance followed by Duncan’s multiple range test was used to evaluate the significance of differences in plant, microbial, and soil variables between different treatments (*p* < 0.05).

## 3 Results

### 3.1 Metabolic functions of functional microbiome

#### 3.1.1 P- and K-solubilizing capacities

**Figure 1:**
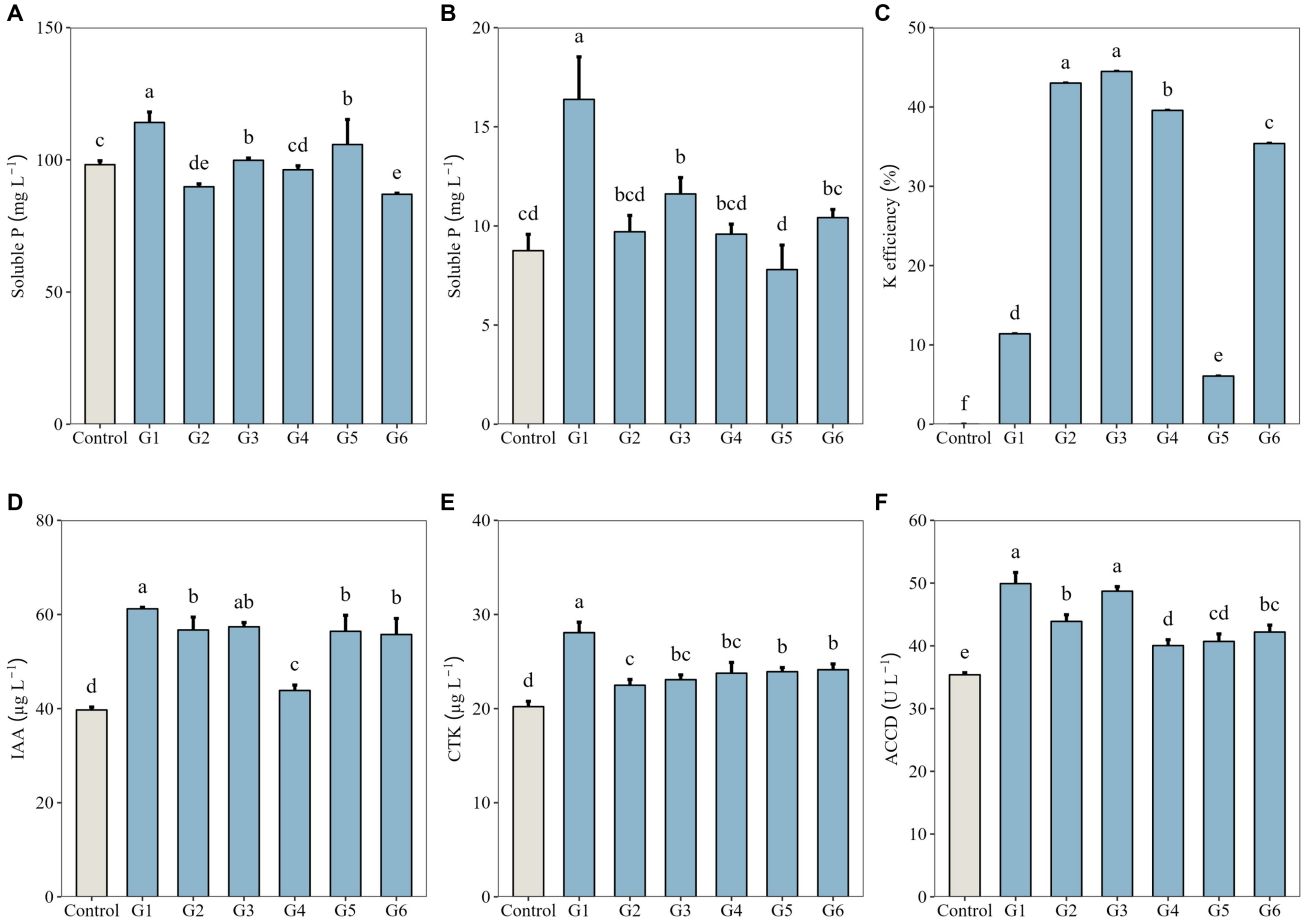
P- and K-solubilizing capacities, phytohormone production, and 1-aminocyclopropane-1-carboxylate deaminase (ACCD) activity of synthetic microbial communities (G1–G6, designed in Table 1). **(A)** Soluble P concentration in Pikovaskaia’s inorganic P medium; **(B)** Soluble P concentration in Monkina’s organic P medium; **(C)** K solubilization efficiency in K solubilization medium; **(D)** Auxin (indole-3-acetic acid, IAA) concentration in combined cultures; **(E)** Cytokinin (CTK) concentration in combined cultures; and **(F)** ACCD activity in combined cultures. Data represent the means ± SD (*n* = 3), and different lowercase letters above error bars indicate significant differences among the treatments (*p* < 0.05).

Among the six SynComs, inoculation with the full community G1 resulted in the highest soluble P concentration (114.14 mg L^−1^) in the inorganic P medium, which was significantly higher than that of the control (98.16 mg L^−1^, *p* < 0.05; Figure 1A). The soluble P concentration in the organic P medium inoculated with G1 (16.38 mg L^−1^) was also the highest, followed by the value of the dropout community G3 (11.61 mg L^−1^, *p* < 0.05) and almost doubling that of the control (8.75 mg L^−1^, *p* < 0.05; Figure 1B). In the K solubilization medium, two dropout communities—G2 and G3—were the most efficient in solubilizing K (43.01 and 44.48%, respectively), whereas G1 exhibited poor solubilization of K (11.40%, *p* < 0.05; Figure 1C).

#### 3.1.2 Phytohormone production and ACCD activity

We additionally analyzed the production of phytohormones by SynComs and their ACCD activity. Both IAA and CTK were detected at significantly higher concentrations in G1–G6 cultures (43.85–61.20 μg L^−1^ and 22.48–28.07 μg L^−1^, respectively) than in the control (*p* < 0.05; Figures 1D,E). The ACCD activity in G1–G6 cultures (40.05–49.91 U L^−1^) was also significantly higher than that of the control (*p* < 0.05; Figure 1F).

### 3.2 Effects of functional microbiome on growth and stress resistance of forage plants

#### 3.2.1 Plant growth under no stress and drought stress conditions

**Figure 2:**
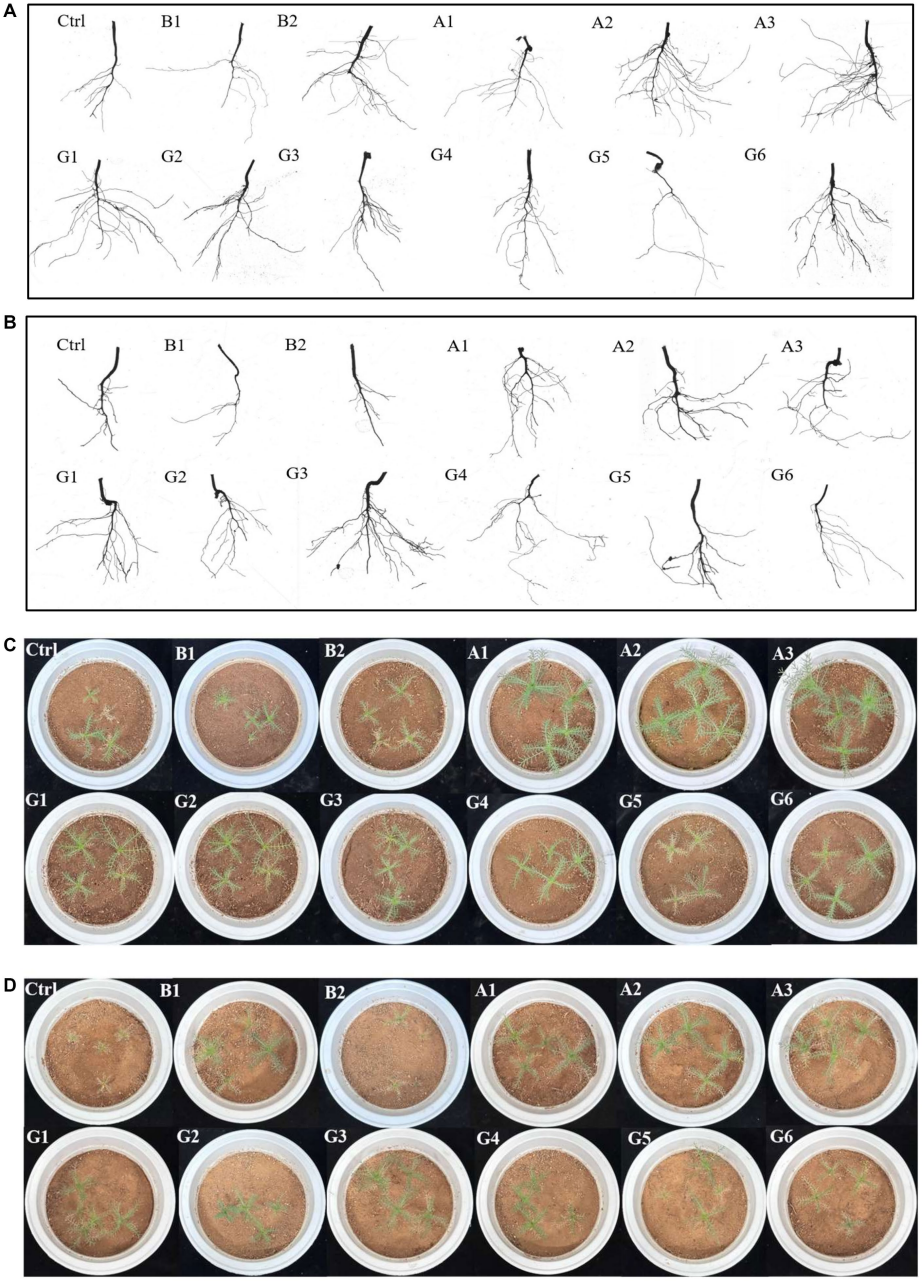
Scanned root images and photographs of *Neopallasia pectinata* plants treated with single strains (B1, B2, and A1–A3) and synthetic communities (G1–G6) under no stress (upper) and drought stress (lower). **(A,B)** Root images and **(C,D)** plant photographs.

**Figure 3:**
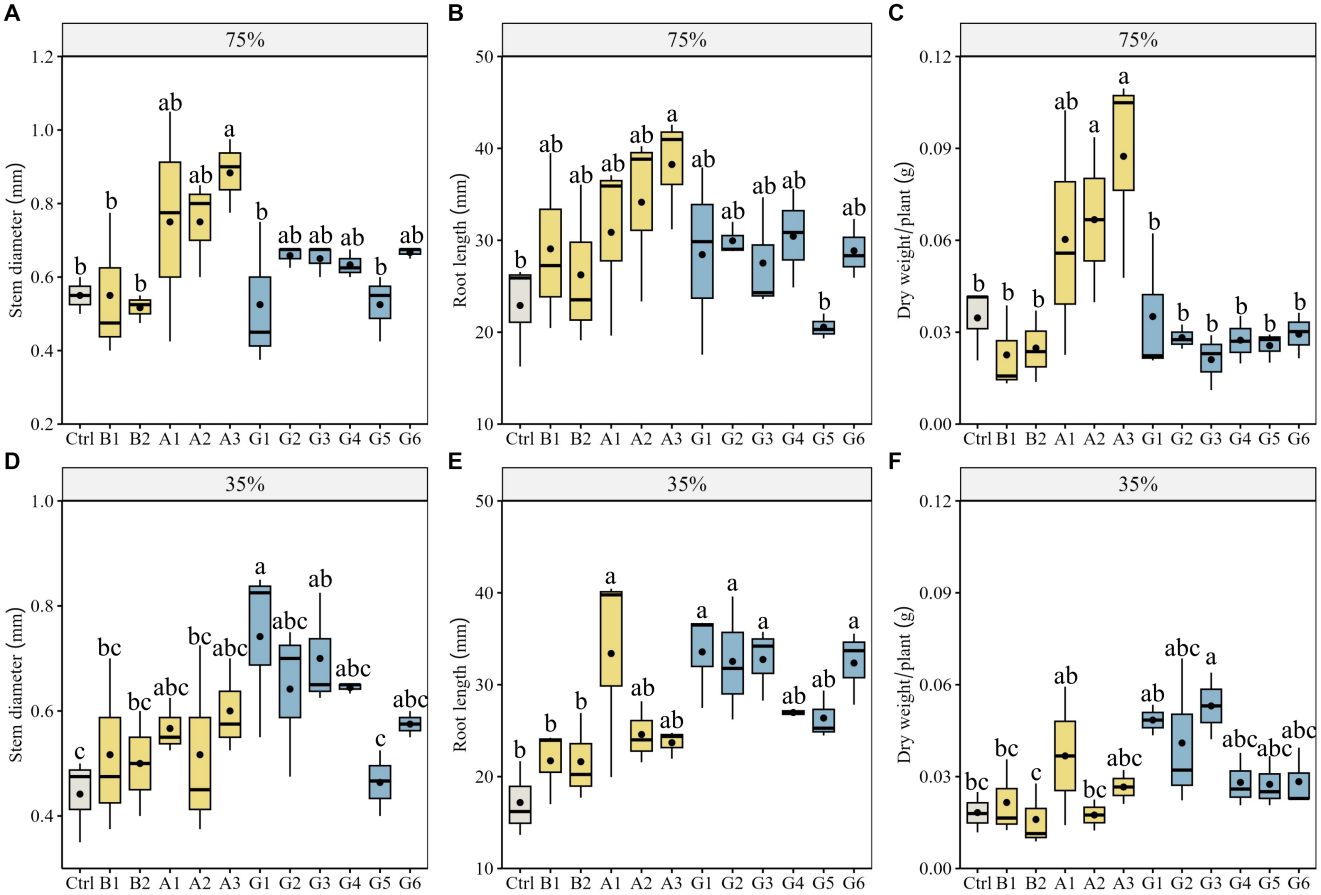
Growth parameters of *Neopallasia pectinata* plants in pots inoculated with single strains (B1, B2, and A1–A3) and synthetic communities (G1–G6) under no stress (75% soil water holding capacity) and drought stress (35% field water holding capacity). **(A,D)** Stem diameter; **(B,E)** Root length, and **(C,F)** Plant dry weight. The horizontal line in the box represents the median of the numerical variable, and the dot represents the mean of the numerical variable (*n* = 3). Different lowercase letters above the error bars indicate significant differences among the treatments (*p* < 0.05).

To ascertain whether drought stress influences the effects of single strains and SynComs on plant growth of *N. pectinata*, we measured stem diameter, root length, and plant dry weight in pots under different water conditions (Figures 2C,D). Under no stress, plants inoculated with strain A3 showed significantly greater stem diameter than controls, with an increase of 60.6% (*p* < 0.05; Figure 3A). The corresponding increase in root length and plant dry weight of A3-inoculated plants reached 67.0 and 143.9%, respectively (*p* < 0.05; Figures 3B,C). The scanned images also showed better root growth in A3 treatment than in other treatments (Figure 2A).

Under drought stress, forage plants showed superior growth performance in SynCom treatments. In particular, plants inoculated with communities G1 and G3 increased by 67.9 and 58.5% in stem diameter, respectively, compared with controls (*p* < 0.05; Figure 3D). The stem diameter of G1-inoculated plants was also significantly greater than that of the B1, B2, and A2 single-strain treatments (*p* < 0.05; Figure 3D). Moreover, distinct increases in plant dry weight (G3) were observed for SynCom treatments (Figures 3E,F). The scanned images showed that the number of fibrous roots and root length in the G3 treatment were greater than those in other treatments (Figure 2B). Plants inoculated with the community G1 increased by 95.44% in root length compared with the control (*p* < 0.05; Figure 3E).

#### 3.2.2 Plant stress resistance under drought stress

**Figure 4:**
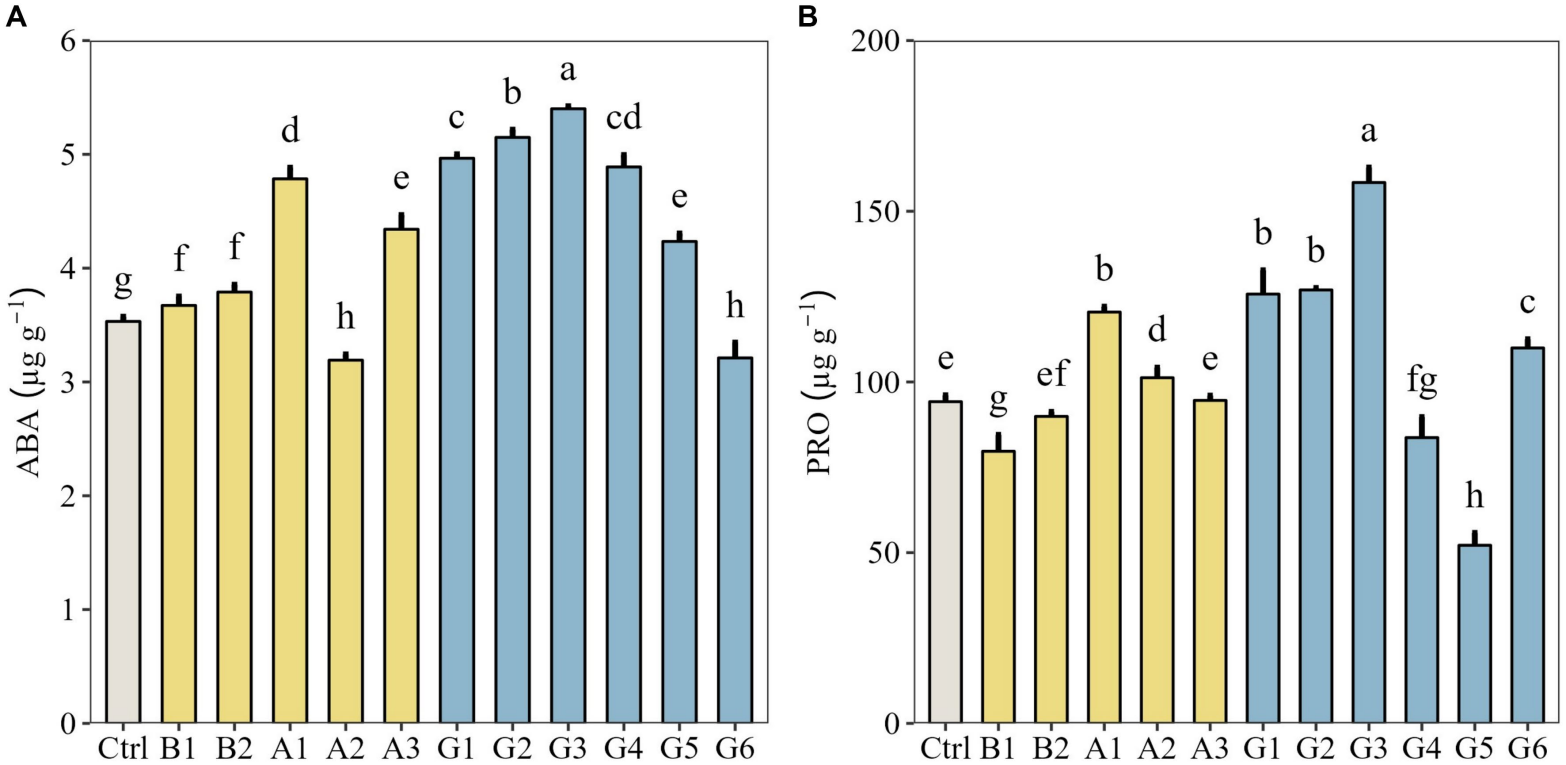
Endogenous hormone biosynthesis and osmoregulator accumulation in *Neopallasia pectinata* plants treated with single strains (B1, B2, and A1–A3) and synthetic communities (G1–G6) under drought stress. **(A)** Abscisic acid (ABA) content and **(B)** proline (PRO) content. Data are presented as the means ± SD (*n* = 3), and different lowercase letters above the error bars indicate significant differences among the treatments (*p* < 0.05).

Next, we measured endogenous hormone biosynthesis and osmoregulator accumulation in plants under drought stress. Except for a single strain (A2) and a SynCom (G6), plants in other microbial treatments all showed significantly higher ABA content than controls (*p* < 0.05). Overall, the highest ABA content was found in G3 treatment (Figure 4A). The PRO content of plants inoculated with G1, G2, and G3 range from 125.75 to 158.42 μg g^−1^, much higher than that of controls (92.23 μg g^−1^; *p* < 0.05). In particular, the PRO content of G3-inoculated plants was significantly higher than that of other treatments (*p* < 0.05; Figure 4B).

#### 3.2.3 Linking functional microbiome to soil nutrients, plant growth, and stress resistance

**Figure 5:**
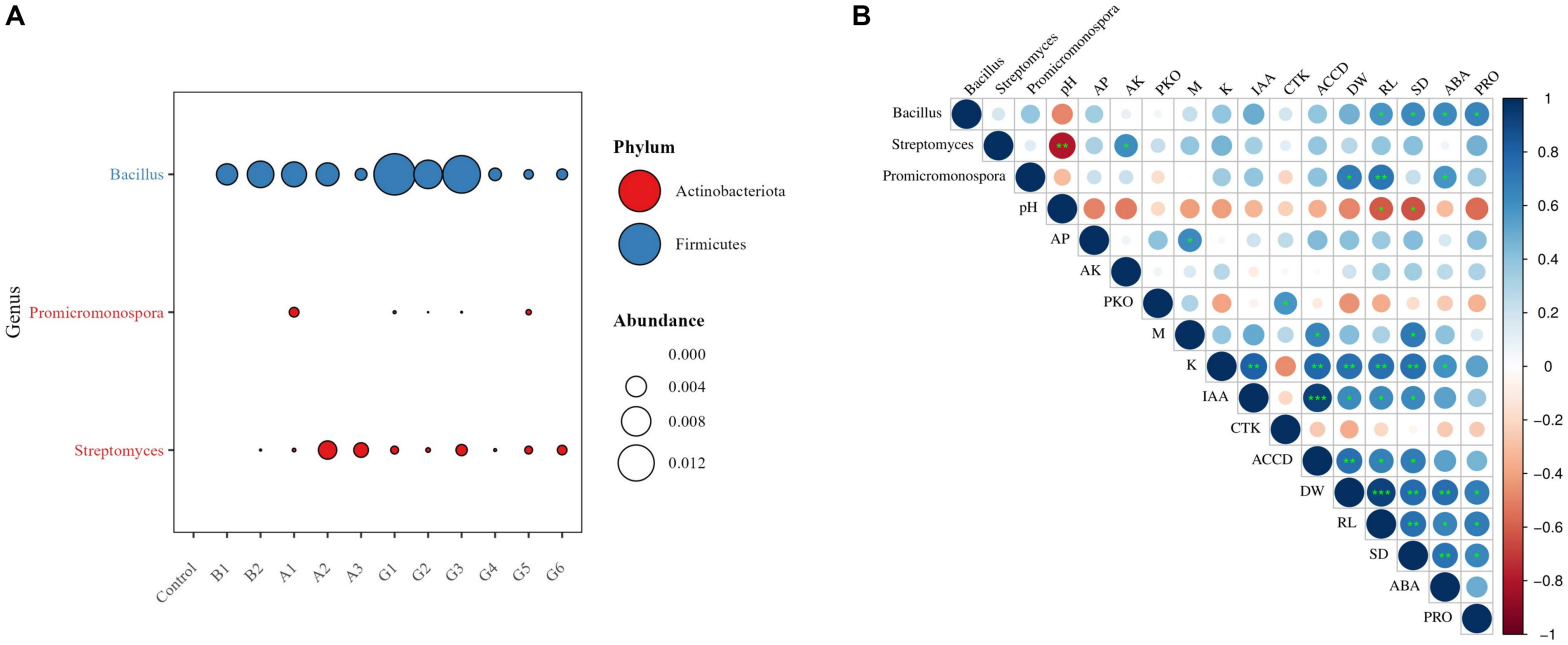
Relationships of functional microbiome, soil properties, and plant traits under drought stress. **(A)** Relative abundance of rhizosphere-colonizing taxa and **(B)** Spearman’s correlations of microbial colonization and metabolite levels with soil properties, plant growth, and stress resistance. Microbial metabolites: PKO, inorganic P-solubilizing capacity; M, solubilized organic P-solubilizing capacity; K, K solubilization efficiency; IAA, indole-3-acetic acid; CTK, cytokinin; ACCD, 1-aminocyclopropane-1-carboxylate deaminase. Soil properties: pH, pondus hydrogenii; AP, available P, and AK, available K. Plant growth parameters: DW, plant dry weight; RL, root length; and SD, stem diameter. Plant resistance traits: ABA, abscisic acid PRO, proline. ^*^p < 0.05, ^**^p < 0.01, and ^***^p < 0.001.

Considering the genus-level taxonomic classification of the five tested strains, we measured rhizosphere colonization by *Bacillus*, *Protomicromonospora*, and *Streptomyces* based on high-throughput sequencing (Figure 5A). Colonization of *Bacillus* was observed across all treatments, with abundances in the range of 0.09%–1.57%. *Promicromonospora* colonized the rhizosphere of plants inoculated with one single strain (A1) and four SynComs (G1–G3 and G5), despite considerably low abundances of 0.0%–0.10%. *Streptomyces* showed successful colonization in all SynCom treatments, with relatively high abundances in the rhizosphere of A2- and A3-inoculated plants (0.32% and 0.21%, respectively). The colonization results were consistent with the strain combinations in the microbial treatments.

Further, we disentangled the relationships between functional microbiome and soil properties, plant growth parameters, and stress resistance traits under drought stress (Figure 5B). *Bacillus* colonization was significantly positively correlated with root length, stem diameter, and plant ABA and PRO contents (*p* < 0.05). *Promicromonospora* colonization was significantly positively correlated with plant dry weight, ABA content, and root length (*p* < 0.05 or 0.01). The P-solubilizing capacity of SynComs was positively correlated with soil Olsen-P content and plant stem diameter (*p* < 0.05). The K-solubilizing capacity of SynComs was significantly positively correlated with plant dry weight, root length, and stem diameter (*p* < 0.01). A significant positive correlation also emerged between the IAA production and ACCD activity of SynComs, and both variables were positively correlated with plant growth parameters (*p* < 0.05 or 0.01).

### 3.3 Effects of functional microbiome on stress resistance of forage plants under different water levels

**Figure 6:**
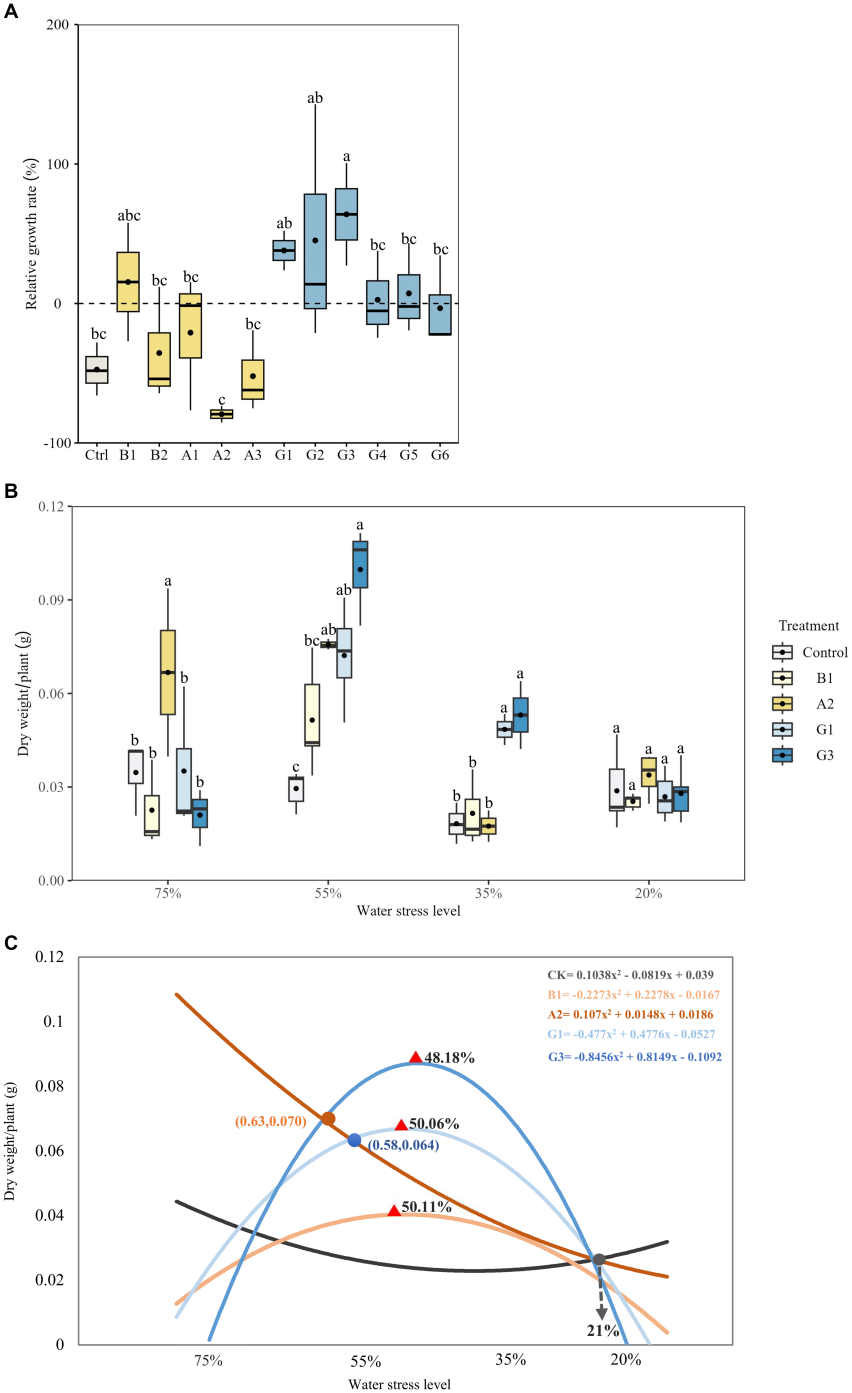
Plant growth of *Neopallasia pectinata* treated with single strains (B1, B2, and A1–A3) and synthetic communities (G1–G6) under different water conditions. **(A)** Box plot showing the relative growth rate of plant biomass under drought stress (35% soil water holding capacity); **(B)** Box plot and **(C)** trend lines of plant dry weight under different drought stress levels. The horizontal line in the box represents the median of the numerical variable, and the dot represents the mean of the numerical variable (*n* = 3). Different lowercase letters above the error bars indicate significant differences among the treatments (*p* < 0.05).

In Experiment I, inoculation with the dropout community G3 resulted in the highest RGR of plant biomass under drought stress, which was significantly higher than that of the control (*p* < 0.05; Figure 6A). Among single-strain treatments, the highest RGR was observed for B1 and the lowest for A2. Accordingly, we selected B1, A2, G1, and G3 to further analyze the effects of functional microbes on plant growth under different water levels (Experiment II).

In terms of plant growth in Experiments I and II, we defined the WHC levels of 75–55%–35–20% as sufficient water–mild stress–moderate stress–extreme stress. Strain A2 performed the best in promoting plant growth under sufficient water conditions, as indicated by significantly higher plant biomass compared with that of other treatments (*p* < 0.05; Figure 6B). Increasing the drought stress level led to continuous decrease in plant biomass in A2 treatment (Figure 6C). In contrast, a notable increase in plant biomass occurred in the G1 and G3 SynCom treatments under mild and moderate drought stress. Plant biomass reached its maximum at 50.1% (G1) and 48.2% (G3) WHC, followed by a decrease afterward. The plant biomass in G1 and G3 treatments remained significantly higher than that of other treatments at 35% WHC (*p* < 0.05). However, there were no significant differences in plant biomass among the treatments at 21% WHC.

## 4 Discussion

Our research innovatively extends the application of SynComs from dominant agricultural settings to the unique environment of desert steppes, highlighting a new avenue in the field of ecological restoration. We specifically focus on the complex interactions between SynComs and drought stress levels, which contributes to a nuanced understanding of microbial function under varying water conditions. We found that drought-stressed *N. pectinata* plants received greater benefits from SynComs than from single bacterial strains that were isolated from the rhizosphere and root endosphere of dominant forage species in desert steppe. Under mild to moderate drought stress, the dropout community G3 (without A2) and the full community G1 exhibited the best effects on plant growth in this dominant forage species. This indicates that manipulation of the functional microbiome is a feasible approach to help forage plants fight stress in desert steppe regions, despite this beneficial effect is limited by a soil moisture threshold.

### 4.1 Functional microbiome boosts plant growth under mild to moderate drought stress

The five strains tested in this study belong to the genera *Bacillus*, *Promicromonospora*, and *Streptomyces*. The SynComs containing four or five of the tested strains were capable of solubilizing mineral nutrients (P and K), secreting phytohormones (IAA and CTK), and exhibiting ACCD activity. Notably, the full community G1 demonstrated the highest P-solubilizing capacity, whereas the dropout community G3 showed excellent performance in both P and K solubilization. The capacities of these SynComs for nutrient solubilization indicate their significant role in enhancing soil fertility, particularly in P-limited environments, including the study area. Microbial P solubilization could contribute to plant growth by increasing soil P supply (Afzal et al., 2022). Additionally, microbial production of IAA and CTK is critical for root development and overall plant health. These microbially derived phytohormones influence root architecture, thereby enhancing the plant’s ability to access soil nutrients. ACCD activity, a key feature observed in the tested strains, plays a vital role in modulating plant stress responses. ACCD activity correlates with a reduction in the endogenous ethylene content in plants, which is crucial for enhancing their resilience to environmental stresses, such as drought or salinity (Van Loon, 2007; De Vries et al., 2018). We also found that the metabolic potassium content of the strain can affect the content of the endogenous hormone ABA (Figure 5B), which is beneficial for plants to adapt to water stress. Water is the most important limiting factor for plant growth in desert steppes, the metabolic functions of these strains are essential for plant growth and particularly favorable for forage plants to resist abiotic stresses.

Our results from high-throughput sequencing indicated successful colonization of *Bacillus*, *Promicromonospora*, and *Streptomyces* in the rhizosphere of *N. pectinata* plants, which is the key to the reciprocal symbiosis between functional microbes and plants (Sasse et al., 2018; Rodriguez et al., 2019). Under drought stress, microbial colonization and metabolite levels correlated with soil pH and nutrient availability (P and K), as well as with plant growth and stress resistance. This provides strong evidence that the functional microbes were likely to solubilize soil mineral nutrients by secreting organic acids and acid phosphatases, which led to improved plant growth and stress resistance (Jeyanthi and Kanimozhi, 2018). Given the low total abundance of functional microbes in the rhizosphere, the colonization result was not ideal. This might be attributable to the small inoculum size of functional microbes (1% of the soil mass) and the late sampling time of rhizosphere soils (20 days after the second inoculation in order not to impair plant growth and to ensure the drought stress level). Despite their low abundance, the functional strains still performed plant growth-promoting functions. A plausible reason is that the strain combination enabled microbial interactions and regulated the overall community structure. Future studies should be conducted to enhance the colonization capacity of the functional strains by increasing the inoculum size or optimizing the inoculation method, as there may be room for improvement in their beneficial effects.

Using a pot experiment, we explored the role of single strains and SynComs in promoting the growth of *N. pectinata* plants under different water conditions. We found that the plant growth-promoting effect of a single strain—A3—was greater than that of other treatments under no stress. The introduction of functional microbes to induce plant growth promotion has been widely recognized and commonly used in agroecosystems for improving crop yield (Wang and Song, 2022). Similar to our findings, other functional strains from natural ecosystems, such as members of the genera *Pseudomonas* and *Klebsiella*, have demonstrated their efficacy in promoting plant growth (Bhojiya et al., 2022). Our study includes strains from the genera *Bacillus*, *Promicromonospora*, and *Streptomyces*, which are also shown to facilitate both aboveground and belowground growth in various crops, such as rice, tomato, and wheat in pot experiments (Niu X. et al., 2017; Chandra et al., 2019; Silva et al., 2020; Ansari et al., 2021). It should be noted that our study particularly emphasizes the role of these strains under varying water conditions, a focus less common in studies involving desert steppes. Additionally, natural ecosystem studies often evaluate microbial function in more complex and diverse environmental settings (De Vries et al., 2018). In contrast, our controlled pot experiment setting allows for a clearer understanding of specific microbial interactions and their direct effects on plant growth, especially under different drought stress levels.

Functional strains often fail in the field when applied singly, and this could be attributable to the inferior adaptability, diversity, and stability of single strains (Souza et al., 2015). In Experiment I, we also found that the effects of single strains on plant growth promotion were inferior to those of SynComs under drought stress. Notably, the dropout community G3 induced an increase in the number of fibrous roots and dry weight of *N. pectinata* plants, which was beneficial for plant adaptation to water-limited environments. Previously, a combination of three bacterial strains (*Pseudomonas putida*, *Pseudomonas* sp., and *Bacillus megaterium*) increased the root biomass of white clover (*Trifolium repens* L.) plants, indirectly improving their tolerance to drought stress (Marulanda et al., 2009). These findings support that consortia of multiple compatible microbes could form stable communities, which outcompete single-species populations under drought stress (Vorholt et al., 2017; Louca et al., 2018). The mechanism of coexistence through microbial interactions is also the key to enhancing plant growth and stress resistance (Raaijmakers and Mazzola, 2012).

Microbial-mediated changes in the root morphological and structural traits of forage plants are an indirect mechanism to induce adaptation to drought stress. Besides, the functional microbiome can stimulate the plants to synthesize resistance-related substances (e.g., phytohormones, osmoregulators), allowing them to directly withstand stressed environments. Under drought stress, G3 was the most efficient to promote ABA and PRO biosynthesis in *N. pectinata* plants. Both substances take part in plant defense against drought stress. Changes in ABA content can enable plant adaptation to drought stress by regulation of stomatal opening and closing (Seki et al., 2007), and PRO plays a role in reducing plant sensitivity to abiotic stresses (Yoshida et al., 2019; Waadt et al., 2022). A previous study found that compared with uninoculated plants, the leaf PRO content of *T. repens* plants increased by 3–4 times following application of mixed cultures composed of arbuscular mycorrhizal fungi and *Bacillus thuringiensis*, which boosted plant drought tolerance (Ortiz et al., 2015). A plausible reason is that when combined into a consortium, functional microbes complement and symbiotize with each other to establish an effective stress response mechanism with plants (Berendsen et al., 2018).

**Figure 7:**
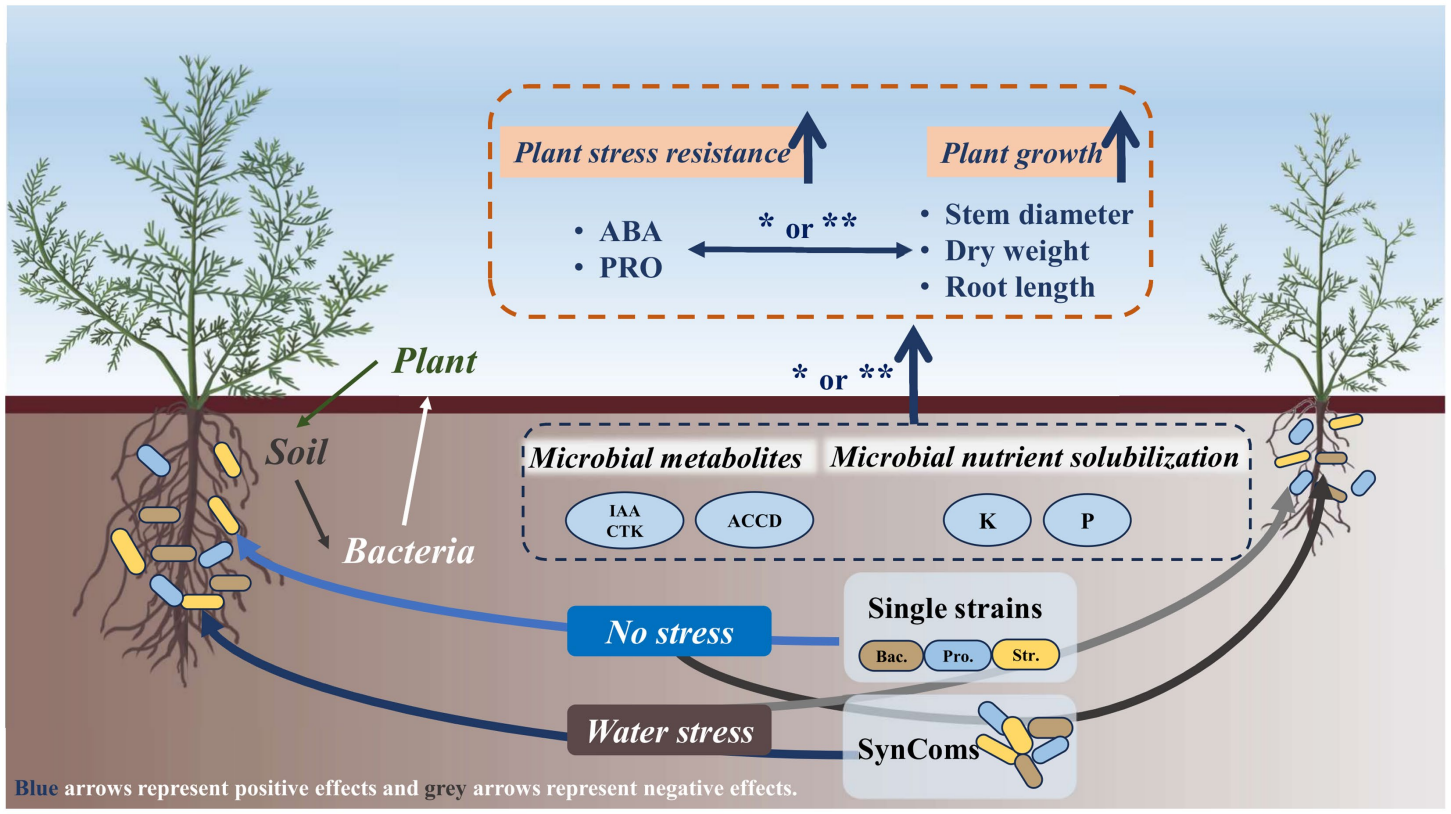
Schematic showing the role of functional bacteria in improving plant growth and stress resistance of *Neopallasia pectinata* in desert steppe. SynComs, synthetic microbial communities; Bac., *Bacillus*; Pro., *Protomicromonospora*; Str., *Streptomyces*; IAA, indole-3-acetic acid; CTK, cytokinin; ACCD, 1-aminocyclopropane-1-carboxylate deaminase; ABA, abscisic acid; PRO, proline. ^*^p < 0.05 and ^**^p < 0.01.

At the experimental site of this study in desert steppe, the application of SynComs is particularly necessary under water limitation. The strains in SynComs can play a positive role in plant growth and stress resistance through direct and indirect effects (Figure 7). Harnessing the functional microbiome in desert steppes is not only an emerging approach to enhance grassland productivity but also a promising contributor to ecological restoration.

### 4.2 Extreme drought stress limits the role of functional microbiome in improving plant growth

Among all microbial treatments, strain A2 proved most efficient in promoting the growth of *N. pectinata* plants under sufficient water conditions (75% WHC), whereas SynComs were more beneficial for drought-stress plants. The advantage of SynComs was prominent under increased drought stress, with G1 and G3 contributing to the maximal plant biomass at ~50% WHC. However, with intensifying stress, there was an inflection point for the difference in the effects of single strains and SynComs compared with controls. In other words, there was a soil moisture threshold for the functional microbes to provide benefits for plant growth, and the positive effects of single strains or SynComs disappeared at 21% WHC. Despite the minimal effects of different drought stress levels on plant biomass (as indicated by only 1.6% variation in the control), the functional microbes still played a positive role in promoting plant growth.

Extreme drought leads to decrease in soil microbial biomass, which means there are fewer microbial drivers of nutrient cycling and other ecological processes in the soil. The capacity of microbes to transform specific nutrients (e.g., N, P, K) may also decline, affecting the utilization of key soil nutrients and hindering nutrient cycling in the plant–soil–microbe system (Fuchslueger et al., 2013; Dijkstra et al., 2015). Additionally, extreme drought causes variation in soil microbial diversity. This is particularly exemplified by a decrease in the proportion and diversity of soil bacteria under extreme drought (Preece et al., 2019). Such structural shifts impair the overall functionality of soil microbiomes and reduces their network stability. Since the strains used in our study were all bacteria, their colonization in the rhizosphere and microbiome structure might have been considerably impacted by extreme drought stress. The results led us to speculate that the application of SynComs can alleviate mild and moderate drought stress in forage plants; persistent and extreme drought stress limits microbial growth, metabolism, and hence function.

Recently, it has been found that an increase in drought intensity and frequency results in decreased resistance and resilience of the natural grassland soil microbiome, with abrupt shifts in microbiome structure and function at ~15% WHC (Cordero et al., 2023). In our case, the functions of single strains and SynComs were already limited under extreme drought stress at 20% WHC. This soil moisture threshold is higher than previously reported, most likely due to the small size of SynComs constructed in the present study. Our SynComs were consortia of four to five functional strains, which proved effective in improving *N. pectinata* plant growth in pots. However, compared with indigenous microbial communities in natural grasslands, a small number of strains have limitations in terms of taxa interactions, symbiotic networks, and ecological functions.

Despite certain controversies regarding the relationship between microbial diversity and ecological function (Jousset et al., 2011; Wagg et al., 2014; Jing et al., 2015), a positive correlation between the two factors has been demonstrated in most relevant studies (Delgado-Baquerizo et al., 2016; Domeignoz-Horta et al., 2020). Rich microbial taxa contribute to ecological function performance, and high microbial diversity positively affects ecosystem multifunctionality by enhancing microbial network complexity (Zhang, 2021). This also suggests that SynComs with higher richness and diversity, as well as more complex network structure, will have greater resistance and resilience to environmental changes. Yet, functional microbes capable of promoting plant growth and stress resistance should not be simply combined during subsequent development and functional studies of SynComs. Rather, appropriate taxa selection and network construction should be taken into account. Increasing the complexity of microbial community structure may also play a vital role in enhancing plant resistance to drought stress.

In this study, the positive effect of SynComs on *N. pectinata* plants vanished under extreme drought stress. Nevertheless, G1 and G3 still conferred substantial benefits on plant growth under mild to moderate drought stress, as indicated by improved plant biomass. Rational application of these two SynComs (especially G3) can provide a useful tool to increase grassland productivity in desert steppes.

## 5 Conclusion

This study demonstrated the effectiveness of the functional microbiome of forage plants in enhancing the growth and drought stress resistance of *N. pectinata*. SynComs were developed with five functional strains from the rhizosphere and root endosphere of dominant forage species in desert steppe. These SynComs leveraged specific metabolic functions—P and K solubilization, IAA and CTK secretion, and ACCD activity—to bolster plant growth and adapt to drought stress. Mild to moderate drought stress boosted the plant growth-promoting effects of SynComs on *N. pectinata*, but there was a soil moisture threshold (21% WHC) for the benefits of SynComs under extreme drought stress.

Our findings underscore the promising application of SynComs in improving forage resilience and growth, particularly under drought stress in desert steppe. The translation of these microbial benefits to natural ecosystems and their stability across various environments remain to be validated. Future research should focus on refining the *in situ* application of SynComs, exploring their effectiveness and stability across grassland ecosystems, and unraveling the mechanisms that underpin their universal benefits for diverse plant species.

## Data availability statement

The datasets presented in this study can be found in online repositories. The names of the repository/repositories and accession number(s) can be found at: NCBI—PRJNA1066100.

## Author contributions

HG: Writing – review & editing. ZH: Writing – original draft. WC: Writing – review & editing, Methodology. AX: Writing – review & editing, Resources. SZ: Writing – review & editing, Methodology. WW: Writing – review & editing, Investigation. HH: Investigation, Writing – review & editing. HL: Writing – review & editing.
