# Supplementary material for: Mild to moderate drought stress reinforces the role of functional microbiome in promoting growth of a dominant forage species (Neopallasia pectinata) in desert steppe
Source: Front Microbiol. 2024 May 22;15:1371208. doi: 10.3389/fmicb.2024.1371208 (PMC11150836; doi:10.3389/fmicb.2024.1371208)
Supplement: Supplementary file 1 [file Data_Sheet_1.PDF]

Supplementary material for

Mild to moderate drought stress reinforces the role of functional microbiome in promoting growth of a dominant forage species (*Neopallasia pectinata*) in desert steppe

Hui Gao[1†], Zhenzhen Huang[1†] Weiwei Chen[1], An Xing[1], Shixiang Zhao[1], Weifan Wan[1], Haina Hu[1] and Haigang Li[1,*]

[1] Inner Mongolia Key Laboratory of Soil Quality and Nutrient Resources, Key Laboratory of Agricultural Ecological Security and Green Development at Universities of Inner Mongolia Autonomous Region, Inner Mongolia Agricultural University, Hohhot 010018, China.

*** Correspondence:**
Haigang Li, Inner Mongolia Key Laboratory of Soil Quality and Nutrient Resources, Key Laboratory of Agricultural Ecological Security and Green Development at Universities of Inner Mongolia Autonomous Region, Inner Mongolia Agricultural University, 29 Erdos East Street, Saihan District, Hohhot 010018, China

Email: haigangli@imau.edu.cn

S1. Moisture gradients definition pre-experiment:

Based on previous studies pre-experiment was conducted, and different moisture gradients were set, including soil water holding capacity (WHC) of 75%, 55%, 35%, and 20%. Each treatment had four replications.

Air-dried soil (1.00 kg) was filled into surface-disinfected pots with an upper diameter of 16 cm, a lower diameter of 9 cm, and a height of 10 cm. Plump seeds were selected(eight per pot). All pots were placed in an artificial climate chamber and incubated for 7 d under the following conditions: temperature, 25°C; illumination time, 8:00–20:00; and light intensity, 400–600 μmol/(m^2 s). Then, four seedlings with uniform growth were established in each pot and water according to different moisture gradients. Plants were harvested after 45 d of incubation and samples were collected to measure plant dry weight.

Fig. S1 Experimental images(a) and plant dry weight(b) of potted plants with different moisture gradients.

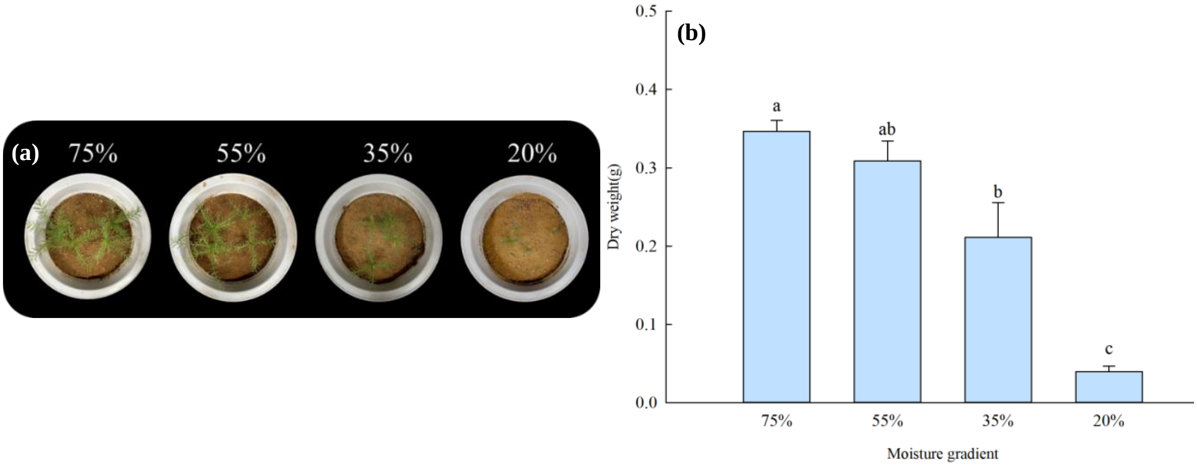

Note: Different lowercase letters above the error bars indicate significant differences among the treatments(n=4) ($P < 0.05$).
